# Effects of interventions for preventing road traffic crashes: an overview of systematic reviews

**DOI:** 10.1186/s12889-021-12253-y

**Published:** 2022-03-16

**Authors:** Ronald Fisa, Mwiche Musukuma, Mutale Sampa, Patrick Musonda, Taryn Young

**Affiliations:** 1 Department of Epidemiology and Biostatistics, The University of Zambia, School of Public Health, Ridgeway Campus, Nationalist Road, Lusaka, Zambia; 2 Centre for Intervention Science in Maternal and Child health (CISMAC), Centre for International Health (CIH), University of Bergen, Bergen, Norway; 3 Centre for Evidence-based Health Care (CEBHC), Department of Global Health, Stellenbosch University, Cape Town, South Africa

**Keywords:** Overview, Road traffic crashes, Systematic review, Interventions

## Abstract

**Background:**

Road traffic crashes (RTCs) are among the eight-leading causes of death globally. Strategies and policies have been put in place by many countries to reduce RTCs and to prevent RTCs and related injuries/deaths.

**Methods:**

In this review, we searched the following databases Ovid Medline, Embase, Cochrane Database of Systematic Reviews, Epistemonikos, Web of Science, and LILACS for reviews matching our inclusion criteria between periods January 1950 and March 2020. We did not apply language or publication restrictions in the searches. We, however, excluded reviews that focused primarily on injury prevention and reviews that looked at crashes not involving a motor vehicle.

**Results:**

We identified 35 systematic reviews matching our inclusion criteria and most of the reviews (33/35) included studies strictly from high-income countries. Most reviews were published before 2015, with only 5 published between 2015 and 2020. Methodological quality varied between reviews. Most reviews focused on enforcement intervention. There was strong evidence that random breath testing, selective breath testing, and sobriety checkpoints were effective in reducing alcohol-related crashes and associated fatal and nonfatal injuries. Other reviews found that sobriety checkpoints reduced the number of crashes by 17% [CI: (− 20, − 14)]. Road safety campaigns were found to reduce the numbers of RTCs by 9% [CI: (− 11, − 8%)]. Mass media campaigns indicated some median decrease in crashes across all studies and all levels of crash severity was 10% (IQR: 6 to 14%). Converting intersections to roundabouts was associated with a reduction of 30 to 50% in the number of RTCs resulting in injury and property damage. Electronic stability control measure was found to reduce single-vehicle crashes by − 49% [95% CI: (− 55, − 42%)]. No evidence was found to indicate that post-license driver education is effective in preventing road traffic injuries or crashes.

**Conclusion:**

There were many systematic reviews of varying quality available which included studies that were conducted in high-income settings. The overview has found that behavioural based interventions are very effective in reducing RTCs.

**Supplementary Information:**

The online version contains supplementary material available at 10.1186/s12889-021-12253-y.

## Background

### Description of the problem

The World Health Organization (WHO) defines a Road Traffic Crash (RTC) as a collision involving at least one vehicle in motion on a public or private road that results in at least one person being injured or killed [1]. Road traffic crashes can result in property damage, injury, or loss of life. A road traffic injury (RTI) is defined by the World Health Organization (WHO) as “a fatal or non-fatal injury incurred as a result of a collision on a public road involving at least one moving vehicle” [2]. Not all RTCs result in injuries however, the latter cannot precede the former.

It is the eighth leading cause of death for all age groups surpassing HIV/AIDS, tuberculosis, and diarrhoeal diseases [3]. Road traffic crashes now represent the eighth leading cause of death globally. The WHO reports that about 1.24 million people die on the roads annually, with 20-50 million sustaining non-fatal injuries [2]. Globally, RTIs are reported as the leading cause of death for children and young adults aged 5–29 years and are among the top three causes of mortality among people aged 15–44 years. More than 85% of the global deaths due to injuries occur in low and middle-income countries (LMICs) consuming substantial health sector resources [4]. The WHO indicates that RTIs cause considerable economic losses to victims, their families, and nations. These losses arise from the cost of treatment (including rehabilitation and incident investigation) as well as reduced/lost productivity (e.g. in wages) for those killed or disabled by their injuries as well as family members who need to take time off work (or school) to care for the injured. Road traffic deaths and injuries are a major but neglected public health challenge that requires concerted efforts for effective and sustainable [4].

Many countries have put forward strategies and policies to curb RTCs to help prevent deaths and injuries. For example, the vision on sustainable safety was developed in 1992 in the Netherlands. In March 2000, the Government of the United Kingdom set out its strategy for improving road safety over the next decade in Tomorrow’s roads: safer for everyone [5]. More recently, in March 2010 the United Nations General Assembly resolution 64/255 proclaimed a Decade of Action for Road Safety 2011–2020 intending to stabilize and then reduce the forecasted level of road traffic fatalities around the world by increasing activities conducted at national, regional and global levels [6]. With the burden of RTCs occurring in LMICs, the Bloomberg Initiative for Global Road Safety (BIGRS) 2015-2019 program is a recent initiative implemented in some LMICs. The program seeks to reduce fatalities and injuries from road traffic crashes in LMICs by strengthening road safety legislation at the national level and implementing proven road safety interventions at the city level [7].

### Description of interventions

The high incidence of RTCs worldwide (HICs and LMICs) has led to the implementation of preventive interventions. Interventions aimed at the reduction of RTCs can be described as a coherent, organized, structured set of objectives and activities implemented to eliminate adverse events related to the use of roads [8]. Interventions for the prevention of RTCs can be tailored for motorists, pedestrians, cyclists, and all other road users. Many systematic reviews exist that attempt to answer the question of whether or not these interventions are effective in reducing RTCs around the world. The reduction of RTCs in different sub-populations has also been the focus of interest in some systematic reviews. A brief search of systematic reviews suggests that legislation is the most common intervention evaluated with the best outcomes when combined with strong enforcement initiatives or as part of a multifaceted approach [9, 10]. Other reviews suggest that graduated driver licensing (GDL) and interventions aimed at improving pedestrian and cyclist visibility as well as area-wide traffic calming has been effective when implemented with concerted efforts [11–13].

### Why is it important to do this assessment of systematic reviews?

Between 2013 and 2016, no reductions in the number of road traffic deaths were observed in any low-income country, while some reductions were observed in 48 middle- and high-income countries [3]. The abundance of different interventions that have been implemented and reported to have a positive effect coupled with the continued increase in the incidence of RTCs, illustrates the need to comprehensively assess and describe the evidence from systematic reviews and the quality of these reviews to identify effective interventions on one hand and gaps in the evidence base on the other hand.

**Fig. 1:**
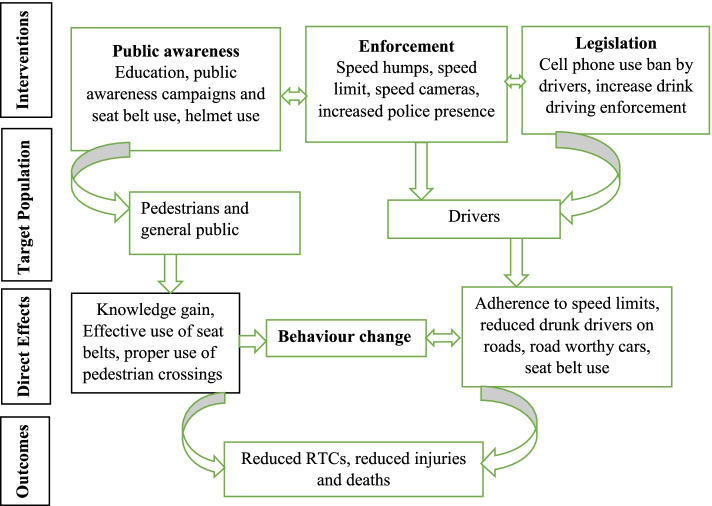
A logic model; Interventions aimed at reducing road traffic crashes around the world which include interventions at individual and organisational level

Interventions for preventing RTCs fall into various categories which include legislation, enforcement, public awareness/education, driver education, and speed control measures such as speed cameras. The logic model below (Fig. 1) shows existing interventions and their target population. These interventions can be targeted at different individuals/groups; Drivers, Riders (motorcyclists & cyclists), Pedestrians, Passengers, all road users, and non-motorized vehicles (hand carts). William Haddon [14] developed a matrix that identifies risk factors before the crash, during the crash, and after the crash, with the person, vehicle, and environment. Since the interventions are a deterrent measure of RTCs, in this overview of systematic reviews, the focus will be on the before the crash stage.

## Methods

The objective of this overview of systematic reviews is to describe the evidence and quality of existing systematic reviews. The main objective of this overview was to summarize the available evidence worldwide from systematic reviews that focused on interventions that have been put in place to reduce RTCs.

### Search methods for identification of systematic reviews

This overview focused on systematic reviews of interventions that aimed at reducing RTCs and subsequently RTIs. To identify the reviews, an information specialist conducted searches in Medline Ovid, Embase Ovid, Web of Science, Epistemonikos (which includes Cochrane Database of Systematic Reviews), Pubmed, EMBASE, The Cumulative Index to Nursing and Allied Health Literature (CINAHL). We further searched PsycINFO, LILACS (Literatura Latinoamericana y del Caribe en Ciencias de la Salud), Database of Abstracts of Reviews of Effects (DARE), and the Campbell Collaboration online library using relevant search terms (See additional Table 11 for the search strategy used). The details of the search strategy including the mesh terms are given in Additional file 2. Reference lists of systematic and related reviews were also searched to find additional potentially eligible studies. All systematic reviews published between 1950 and March 2020, which included Cochrane and non-Cochrane reviews, were considered. The search was again conducted in September 2021 during the revision of the manuscript to identify new reviews. We did not find any review that focused of interventions to prevent RTCs. The searches were not restricted by language or publication status.

### Criteria for considering reviews for inclusion

This overview considered all systematic reviews that focused on interventions and measures that have been put in place to reduce RTCs around the world. Reviews that were included satisfied our definition of a systematic review according to Antman and Oxman. The reviews to be included should clearly state the objectives, searched for studies on two or more databases including grey literature/unpublished work, extracted data should have been analysed, and a risk of bias assessed for each study with results presented appropriately [15–17]. For any systematic review to be included, the PICO component must be satisfied (population, intervention, comparator, and outcome). The population in these reviews included people who use roads such as drivers, pedestrians, cyclists etc. Interventions included sobriety check points, lowering Blood alcohol content, road expansions, mass media campaigns among others and comparators included areas/sections where there was no intervention. The outcome of the overview was road traffic crashes (RTC).

### Systematic review selection, data extraction and management

**Fig. 2:**
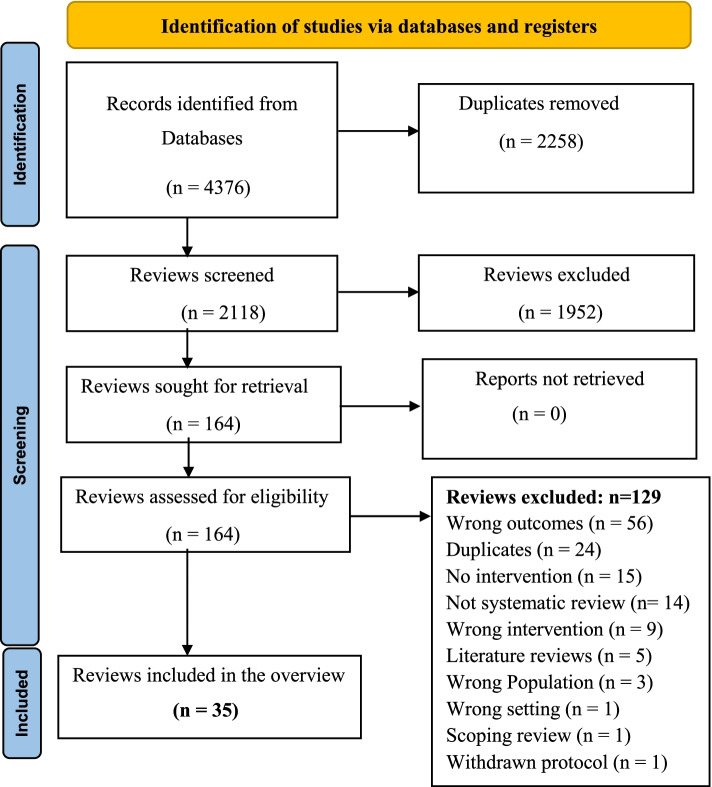
PRISMA Flow diagram; selection of relevant systematic reviews meeting the inclusion criteria for the overview

Titles and abstracts were examined independently by two reviewers and full text articles of the selected titles and abstracts were retrieved for further scrutiny. The full texts were independently assessed using the pre-specified eligibility criteria. A final decision on the included studies was made and the data extraction phase began. Conflicts during the screening process were resolved by a third reviewer. A PRISMA flow diagram (Fig. 2) shows the screening process of articles up to the final number of reviews which were included in the overview.

A checklist of items to consider when extracting the data from the systematic reviews was created. The checklist included items on the methods used in each systematic review, the interventions considered in the review and results which were obtained in each systematic review. We then summarized data from the systematic reviews in the table of characteristics of included systematic reviews. The methodological quality of the systematic reviews was assessed using AMSTAR 2 [18], a critical appraisal tool for systematic reviews that include randomized or non-randomized studies of health care interventions. It consists of 16 domains which must be answered with a yes, partial yes, N/A or a no. The domains are given in the following table (Table 1).

**Table 1:** AMSTAR 2 Domains

**ITEM 1:** Did the research questions and inclusion criteria for the review include the components of PICO (Population, Intervention, Comparison and Outcome)? **ITEM 2:** Did the report of the review contain an explicit statement that the review methods were established prior to the conduct of the review and did the report justify any significant deviations from the protocol? **ITEM 3:** Did the review authors explain their selection of the study designs for inclusion in the review? **ITEM 4:** Did the review authors use a comprehensive literature search strategy? **ITEM 5:** Did the review authors perform study selection in duplicate? **ITEM 6:** Did the review authors perform data extraction in duplicate? **ITEM 7:** Did the review authors provide a list of excluded studies and justify the exclusions? **ITEM 8:** Did the review authors describe the included studies in adequate detail? **ITEM 9:** Did the review authors use a satisfactory technique for assessing the risk of bias (RoB) in individual studies that were included in the review? **ITEM 10:** Did the review authors report on the sources of funding for the studies included in the review? **ITEM 11:** If meta-analysis was performed did the review authors use appropriate methods for statistical combination of results? Includes a. RCT and b. NRSI (non-randomised studies) **ITEM 12:** If meta-analysis was performed, did the review authors assess the potential impact of RoB in individual studies on the results of the meta-analysis or other evidence synthesis? **ITEM 13:** Did the review authors account for RoB in individual studies when interpreting/ discussing the results of the review? **ITEM 14:** Did the review authors provide a satisfactory explanation for, and discussion of, any heterogeneity observed in the results of the review? **ITEM 15:** If they performed quantitative synthesis did the review authors carry out an adequate investigation of publication bias (small study bias) and discuss its likely impact on the results of the review? **ITEM 16:** Did the review authors report any potential sources of conflict of interest, including any funding they received for conducting the review?	

### Data analysis

This overview was a descriptive study that aimed at describing all systematic reviews that focused on interventions aimed at reducing the incidence of RTCs. The overview also identified interventions that were effective in reducing RTCs as well as ineffective interventions. We reported the type of interventions that assessed in the included systematic reviews, we also stratified the included systematic reviews according to the economic status of the countries in which the reviews were conducted. For example, we reported the number of systematic reviews which were conducted in LMICs. We further examined potential overlap between included systematic reviews.

## Results

This overview included systematic reviews that had interventions to prevent road traffic crashes around the globe. The objective was to describe and summarize findings from existing reviews. The different intervention which were assessed are included in Table 2.

**Table 2:** Summary of included systematic reviews

Category	Number of reviews	Systematic reviews
Driver education	4	Curry et al., 2015 [19], Ker et al., 2005 [20], Korner-Bitensky et al., 2009 [21], Roberts and Kwan, 2001 [22]
Enforcement	11	Aeron-Thomas and Hess, 2005 [23], Blais and Dupont, 2005 [24], Bunn et al., 2003 [12], Elder et al., 2002 [25], Erke, 2009 [26], Erke et al., 2009 [27], Goss et al., 2008 [28], Høye, 2014 [29], Peek-Asa, 1999 [30], Pilkington and Kinra, 2005 [31], Wilson et al., 2010 [32]
Legislation	3	Foss and Evenson, 1999 [33], Russell et al., 2011 [34], Zwerling and Jones, 1999 [35]
Mixed Interventions	3	Aguilera et al., 2014 [36], Bergen et al., 2014 [37], Lefio et al., 2018 [38]
Other interventions	3	Ditter et al., 2005 [39], Kwan and Mapstone, 2006 [40], Subzwari et al., 2009 [41]
Public Awareness	5	Duperrex et al., 2002 [42], Elder et al., 2005 [43], Elder et al., 2004 [44], Phillips et al., 2011 [45], Yadav and Kobayashi, 2015 [46]
Structural improvement	4	Beyer and Ker, 2009 [47], Elvik, 2003 [48], Elvik, 2017 [49], Fu et al., 2016 [50]
Vehicle Design Improvement	2	Elder et al., 2011 [51], Erke, 2008 [26]

### Results of the search

The search for the overview was conducted in December 2019 and identified 4376 systematic reviews, and of these, 2258 reviews were excluded as they were duplicates. A total of 2118 abstracts were screened and 1952 studies were deemed irrelevant and were excluded. One hundred and sixty-four (164) full text studies were assessed for eligibility and out these studies, 129 were excluded. The reasons for exclusion included wrong outcomes, wrong intervention, wrong setting and wrong patient population (Fig. 2). Thirty-five (35) systematic reviews were included in this overview. The interventions in these reviews were then classified into different categories such as enforcement, driver education, structural improvement, legislation, public awareness, improvement of vehicle design, mixed intervention and other intervention.

### Description of included reviews

Thirty-five (35) of the systematic reviews in the overview included studies from high-income countries. Only one review [50] included studies from high-income, upper-middle and low-income countries. One systematic review did not find any studies that met the inclusion criteria despite the search being updated twice [41, 52, 53]. Most reviews were published prior to 2015, only 5 published between 2015 and 2020.

### Enforcement results

Eleven systematic reviews looked at enforcement of laws that help reduce RTCs (Table 3 in Additional file 3) [12, 23–32, 54, 55]. Some of these enforcement strategies included police patrols, sobriety check points, speed cameras and speed control measures such as humps. Little overlap existed between these systematic reviews. After conducting quality assessment of the reviews using AMSTAR 2 (See additional file 1), results indicated that 4 reviews were of critically low quality [25, 26, 30, 31], 3 were of low quality [24, 27, 54] and 4 were of moderate quality [23, 28, 29, 32].

The reviews found different results depending on the type of intervention. Reviews such as one done by Aeron-Thomas and Hess [23] conducted in high-income countries (Australia, Singapore, USA) on red-light cameras (RLCs) on RTCs found that that RLCs reduced total casualty crashes though the investigators indicated that there was limited evidence available regarding the reduction of right-angle or rare-end crashes. Contrary to these findings of reduction in motor vehicle crashes by RLCs, a review by Erke [26] where the effects of RLCs on crashes was investigated in high income countries, the findings for RLCs were rather unfavourable. Results from this overview indicated that right-angle collisions were reduced by about 10%, whereas the rear-end collisions increased significantly increased by 40% and the overall effect of RLCs on all types of crashes is an increase by about 15% (Erke, 2009). Further, another systematic review by Pilkington and Kinra [31] assessed the effectiveness of speed cameras in reducing road traffic collisions and related casualties. The review found that speed cameras consistently reduced road traffic collisions effectively as an intervention. In the same line of interventions but with regard to speed cameras, Wilson et al., [32] conducted an assessment to investigate whether the use of speed cameras on the roads reduces incidence of speeding, road traffic crashes, injuries and deaths. In this review, findings showed that speed cameras were an effective intervention for reducing RTIs and deaths. There was however, a weak level of evidence as only 12 of the 35 studies included in the review were of high quality. Most of these reviews (23/35) were of poor quality [31].

Other interventions which fall under enforcement included police intervention, assessing these interventions indicated a reduced number of crashes. This reduction however varied between 23 and 31% [24]. The review further found that all the types of police interventions reviewed, such as enhancing police controls were effective in reducing RTCs and as a result improved road safety.

Traffic calming has been used as one of the interventions to reduce RTCs. In this vain, a review by Bunn et al. [12] consisting of controlled before-after studies revealed that area-wide traffic calming schemes may have the potential to reduce road traffic deaths and injuries. However, there was no evidence that traffic calming schemes prevented pedestrian-motor collisions. One of the important effects of traffic calming schemes is to reduce the speed of traffic and by doing this, we reduce the likelihood of injury in the event of a collision.

Other interventions focused on reduction of drinking and driving which is one of the common factors documented to be associated with RTCs. Interventions in this category included sobriety check points and this was assessed for effectiveness. Findings from one review by Elder [25] indicated that there was a strong evidence that both random breath testing (RBT) and selective breath testing (SBT) sobriety check points were effective in reducing alcohol-related crashes and associated fatal and nonfatal injuries. The results of this review were consistent with results from other reviews such as those by Peek-Asa [30] and Erke et al., [27] on sobriety check points. The review by Erke [27] revealed that the overall effect of driving under influence (DUI) checkpoints on the number of crashes had an estimated reduction of 17% [CI: (− 20; − 14)] and when controlled for publication bias, the estimated reduction in RTCs was 14%. The reviews which fall under this category are summarized in Table 3 below.

**Table 3:** Description of included systematic reviews: Enforcement

Review first author, date of publication	Date of search to identify studies for this review	Interventions	Participants	Number of included studies (type of study design)	Geographic area	Outcomes
Aeron-Thomas 2005 [23]	June-02	Cameras used at intersections to detect red‐light violators	All road users, Intersections and areas assigned red-light cameras.	10 (controlled before and after)	HIC (USA, Australia, Singapore)	Road traffic casualties and crashes Red-light violations
Blais 2005 [24]	Not stated	Tough police interventions (random breath testing, sobriety checkpoints, random road watch, photo-radar)	Drivers of all motorised vehicles	10 (before and after studies)	High-income countries	Decrease in accidents
Bunn 2003 [12]	Jan-08	Vertical and horizontal shifts in traffic (e.g. road humps, speed cushions, raised crosswalks. Reduced speed limit zones.	Areas covering a number of different streets, including residential and main roads, treated with traffic calming measures	22 (controlled before-and-after studies)	HIC (Germany, UK, Australia, Netherlands, Denmark, Japan, and Spain)	Road traffic crashes. Pedestrian-motor vehicle collisions
Goss 2008 [28]	Not stated	Police patrols that target alcohol-impaired driving	All drivers using public roads	32 (RCTs, controlled before and after, controlled interrupted time-series)	High-income countries	Alcohol-impaired driving
Elder 2002 [25]	Not stated	Sobriety checkpoints	Drinking drivers	17 (interrupted time-series, before and after)	High-income countries	Reducing alcohol-involved crashes
Erke 2009 [26]	Not stated	Red light cameras	Drivers of all motorised vehicles	21 (before and after, time series)	High-income countries	Reduction of crashes
Erke 2009 [27]	Not stated	Drink driving check-points	Drivers of all motorised vehicles	40 (before and after, interrupted time series)	HIC (Australia, UK, USA, Canada, New Zealand)	Reduction of crashes involving alcohol
Hoye 2014 [29]	Not stated	Speed cameras and section control (point-to-point speed cameras)	Drivers of all motorised vehicles	9 (before and after studies)	HIC (Australia, Belgium, Norway, Sweden, Italy, USA, Scotland)	Total crash numbers
Pilkington 2005 [31]	Feb-04	Speed cameras	Drivers of all motorised vehicles	14 (Controlled before and after, before and after without control, time-series analysis)	HIC (UK, Canada, Australia, New Zealand, Norway)	Road traffic collisions, Injuries, and deaths.
Peek-asa 1999 [30]	1997	Random Alcohol Screening in Reducing Motor Vehicle Crash Injuries	Drivers of all motorised vehicles	14 (Ecological studies, interrupted time-series, quasi-experimental time-series)	HIC (USA, Australia)	Random screening significantly reduced crashes and injuries
Wilson 2010 [32]	Mar-10	All automated and semi‐automated methods and systems available for speed enforcement (speed cameras (photo radar) laser and other radar devices as well as ancillary equipment such as road embedded electromagnetic loops.)	Drivers of all motorised vehicles	35 (controlled before‐after trials, interrupted time series studies)	HIC (Australia, Canada, Germany, Denmark, Spain, Finland, England, UK, London, Hong Kong, Netherlands, Norway, New Zealand, USA)	Percentage of speeding drivers above the speed limit The absolute pre/post change in speed or the percentage pre/post change in speed in areas with and without cameras. Duration of speed reduction (i.e. time and distance halos): Crash and injury outcomes

### Public awareness

In this category, five reviews fell under this and assessed public awareness strategies [42–46]. All the reviews included data from high income countries. One of the reviews focused on awareness campaigns targeting pedestrians [42] while the remaining four reviews focused on awareness campaigns which targeted drivers [44–46]. Little overlap was observed between the systematic reviews in this category. After an assessment of the methodological quality of the reviews, we found that three out of the five reviews were critically of low quality [43, 44, 46] and two were of moderate quality [42, 45]. A summary of the methodological quality of all the reviews is included in the attachments (additional file 1).

A review by Dupperex [42] assessed the effectiveness of safety education with respect to pedestrians. The review did not find any significant information on the magnitude of the driver education effectiveness in reducing child injuries. A meta-analysis on road safety campaigns conducted by Phillips, (2011) found that road safety campaigns reduce the numbers of road crashes by 9% [CI: (− 11%; − 8%)]. In the same line Elder [43] focused primarily on the effectiveness of School Based Programs for reducing drinking and driving (DD) behaviour but the review did not find sufficient evidence regarding its effectiveness. Mass media campaigns is one of the strategies which countries have been using in reducing RTCs. A review by Elder [44] found some decrease in crashes across all studies and all levels of crash severity was 13% (IQR: 6 to 14%). Similar findings were obtained by Yadav [46] where he found that studies that evaluated the impact of mass media campaigns independently showed reduction more consistently with a median of 15.1% (28.8, 0). A summary of these reviews is given in Table 4 below.

**Table 4:** Description of included systematic reviews: Public Awareness

Review first author, date of publication	Date of search to identify studies for this review	Interventions	Participants	Number of included studies (types of studies)	Geographic area	Studied Outcomes
Duperrex O, Roberts I, Bunn F 2002 [42]	1999, and updated in May 2003.	Pedestrian safety education and media awareness campaigns	Pedestrians of all ages	15 (Randomized Controlled Trials)	HIC (United Kingdom, England, Australia, Germany, USA, Japan, Canada, Scotland)	• Pedestrian-motor vehicle collisions. **.** Behaviour, attitude and knowledge of pedestrians.
Elder 2004 [44]	December 2002	Mass media campaigns	Drivers	8 (Interrupted time series, before and after)	HIC (USA, New Zealand, Australia)	Reduced alcohol impaired driving and alcohol-related crashes
Elder 2005 [43]	Not stated	School based instructional programs, peer organizations, and social norming campaigns.	Drivers	13 (before and after, group randomized controlled trials, non-randomized trial, interrupted time series)	HIC (Australia, USA, New Zealand)	Reduced drinking and driving and riding with drinking drivers
Phillips 2011 [45]	2008	TV, radio and newspaper adverts, roadside messages,	Drivers	67 (controlled before and after, interrupted time series	HIC (USA, Australia, Denmark, Norway)	Reduced alcohol related crashes
Yadav 2015 [46]	31 December 2013	media campaigns with or without concomitant enforcement activities	Drivers	19 (controlled interrupted time series, uncontrolled interrupted time series, controlled before-after studies)	HIC (USA, Australia, New Zealand)	alcohol-related fatal crashes

### Structural improvement

In structural improvement, we looked at interventions that brought about the change in road network, signage etc. In this category one review assessed the safety effects of street lights [47], another one looked at the effects and efficiency of digital countdown timers [50], and the remaining two reviews focused on the effectiveness of converting intersections into roundabouts [48, 49]. Of the four reviews, only one review by Fu et al. [50] included primary studies from high income, upper-middle and lower-middle countries, while all the other reviews only included primary studies from high income countries. A lot of overlap existed between two studies conducted by Elvik [48, 49] as all the studies used in the systematic review by [48] were also included in the 2017 review. Table 5 below gives systematic reviews which were considered in this category.

**Table 5:** Description of included systematic reviews: Structural improvement

Review first author, date of publication	Date of search to identify studies for this review	Interventions	Participants	Number of included studies (types of studies)	Geographic area	Studied Outcomes
Beyer 2009 [47]	October 2008	Street lights	Streets or groups of streets	17 (controlled before and after studies)	HIC (USA, UK, Australia, Germany)	**.** Slight reduction in crashes **.** Slight reduction in fatal and injury crashes
Elvik 2003 [48]	1997	Roundabouts	Intersections	28 (before and after, comparative study)	HIC (Britain, Sweden, Denmark, Norway, Australia, Netherlands, Switzerland)	**.** Reduction in number of injury and fatal crashes
Elvik 2017 [49]	Not stated	Roundabouts	Junctions	44 (before and after, comparative study, cross-sectional studies)	HIC (Britain, Sweden, Denmark, Norway, Australia, Netherlands, Switzerland, USA, Belgium)	**.** Reduction in number of crashes
Fu 2016 [50]	February 2015	Digital countdown timers	Motorists	14 (cross-sectional studies, before and after studies)	HIC (Taiwan, China, Singapore, Slovenia) MIC (Thailand) LMIC (India)	**.** No reduction in rear-end collision **.** No effectiveness in DCT on intersections.

A review conducted by Beyer [47] evaluated street lighting and prevention of RTIs. This review consisted of controlled before-after studies and the findings suggested that street lighting may prevent road traffic crashes, injuries and fatalities.

Other reviews in this category included Elvik’s 2003 and 2017 [48, 49] studies in which the effects of converting intersections to roundabouts in order to improve on road safety was examined. Elvik found that roundabouts were associated with a reduction of 30 to 50% in the number of injury accidents, and fatal accidents were reduced by 50 to 70% [48]. Similarly, an updated review by Elvik [49], revealed that converting junctions to roundabout was associated with a reduction of fatal accidents. The reduction of fatal accidents was estimated to be of 65%, while a reduction of injury accidents was estimated to be approximately 40%.

Regarding the Digital countdown timers (DCT), a review by Fu [50] found no evidence of DCT being effective on intersections. The author therefore, made no recommendations with regard to installing DCT at signalized intersections as a way of improving road safety and operational efficiency.

### Legislation

Three reviews were included under the legislation category [33–35]. Interventions in these reviews included graduated driver licensing (GDL) programs for teenage drivers [33, 34] and low blood alcohol concentration (BAC) laws among younger drivers [35]. Due to the nature of the interventions, none of the reviews utilized data from a randomized controlled trial (RCT) but all three utilized data from observational studies. All three reviews used primary studies conducted in either in the United States of America, Canada, Australia, or New Zealand. There was little overlap between two reviews Foss and Russel in this category [33, 34]. All the reviews in this category are given in Table 6 below.

**Table 6:** Description of included systematic reviews: Legislation

Review first author, date of publication	Date of search to identify studies for this review	Interventions	Participants	Number of included studies (types of studies)	Geographic area	Studied Outcomes
Foss 1999 [33]	1997	Graduated driver licensing	Teenage drivers	7 (Ecologic mixed study and Ecologic time study)	HIC (New Zealand and USA)	**.** No reduction in motor vehicle crashes, injuries and fatalities
Russel 2011 [34]	May 2009 and September 2009	Graduated driver licensing	Teenage drivers	34 (ecological design, cross-sectional design, controlled before and after,	HIC (USA, Canada. Australia, New Zealand)	**.** Reduction in crash rates of teenage drivers’ **.** Reduction in all crash types
Zwerling 1999 [35]	1997	Low blood alcohol concentration (BAC) laws	Teenage drivers	6 (Before and after study, interrupted time-series)	HIC (Australia, Canada, USA)	**.** Reduced motor vehicle crashes

A review by Foss [33] focused on GDL programme and their effectiveness. The review found that insufficient data on GDL programs to assess their effectiveness in reducing RTCs and crashes. One of the limitations in this review was that there were very few graduate driver licensing programs around the world and thus evidence was limited. However, a latest review done by Russel [34] on GDL found that the program was effective in reducing crash rates of teenage drivers. The GDL was very effective in reducing all crash types, although this is not a common intervention especially in LMIC.

Zwerling [35] focused on blood alcohol content and aimed at investigating if the reduction in the BAC could lead to reduced RTCs and crashes, the law was effective and the number of accidents were seen to be reducing. Reductions in RTCs/crashes ranged from 11 to 33% with a cluster of parameter estimates just under 20% [35].

### Driver education

Four reviews were included in the driver education category [19–22]. Two of the reviews focused on driver education programs targeting teen drivers [19, 22], one focused on retraining of older individuals [21] while another focused on post-license driver education for all drivers, regardless of age (reported in two publications) [20]. All reviews included primary studies from high income countries. Table 7 below gives a summary of included studies in this category.

**Table 7:** Description of included studies: Driver Education results

Review first author, date of publication	Date of search to identify studies for this review	Interventions	Participants	Number of included studies (types of studies)	Geographic area	Studied Outcomes
Curry 2015 [19]	January 2014	Parent-directed teen driving. Supervised practice driving	Drivers below the age of 21	31 (RCT, before and after)	HIC (Israel, USA)	**.** No reduction in Road traffic crashes
Robert and Kwan 2001 [22]	Updated May 2006	School-based driver education	Individuals between the age of 15 to 24 years who had not yet obtained a licence.	3 (RCTs)	HIC (Australia, United States, New Zealand)	**.** No reduction in Road traffic crashes **.** Increase in Road related injuries (fatal and non-fatal) due to teenage driving.
Ker 2005 [20]	October 2005.	Post-licence driver education	Motor vehicle drivers (including motorcyclists) with valid driving licence	24 (RCTs)	HIC (USA, Sweden)	**.** No reduction in Road traffic crashes **.** No reduction in Injury crashes (fatal and non-fatal injuries caused by a crash).
Korner-Bitensky 2009 [21]	January 2008	Skill-specific driver training programs for older individuals	Older drivers training	RCT, Matched pairs Cohort, descriptive study, pre-post study	HIC (Canada)	**.** Driving awareness/knowledge on-road driving behaviour and skills **.** Reduction in crash rates

In a review by Curry [19], findings were that the teens driving interventions only improved parental supervisory behaviours and increased teen driver skill acquisition, however, the intervention did not demonstrate a reduction on teen crashes and resultant injuries. These findings were consistent with Roberts and Kwan [22] who also found no evidence that driver education reduces teenage involvement in road traffic crashes but driver education only leads to early licencing. Roberts and Kwan’s’ research further observed that because driver education encourages earlier licensing, it may lead to a modest but potentially important increase in the number of teenagers involved in road traffic crashes.

A study be Ker [20] quantified the effectiveness of post-licence driver education in reducing road traffic crashes. This review found no evidence that post-licence driver education is effective in preventing RTIs or crashes. The authors indicated that although the results are compatible with a small reduction in the occurrence of traffic offences, there’s no evidence of this being truly effective; and this may be due to selection biases or biases in the included trials. Driver retraining especially older drivers was also looked at by Korner-bitensky [21]. Interventions included in the review were classroom sessions, on road sessions and on road education in comparison with controls. In this review, Korner-bitensky found strong evidence from randomized controlled trials (RCTs) that an educational intervention curriculum versus no intervention was effective in reducing crashes.

### Improvement of vehicle design

Two reviews by Elder et al., and Erke looked at interventions targeted at improving the designs of motor vehicles [51, 56]. The study by Elder looked at the effect of ignition interlocks on reducing alcohol impaired driving and alcohol related crashes [51] while Erke [56] focused on the effect of electronic stability control (ESC) in reducing RTCs. In these two systematic reviews, we found no overlap. Both systematic reviews only included studies from high income countries, as demonstrated in Table 8.

**Table 8:** Description of included studies: Improvement of Vehicle design

Review first author, date of publication	Date of search to identify studies for this review	Interventions	Participants	Number of included studies (types of studies)	Geographic area	Studied Outcomes
Elder 2011 [51]	December 2007	Ignition interlocks	Drivers convicted of driving while impaired	15 (RCT, cohort, before and after study)	HIC (Canada, USA, Sweden)	**.** No reduction in Alcohol-related crashes
Erke 2008 [56]	Not stated	Electronic stability control (ESC)	Vehicles with and without ESC	8 (Before and after studies, case-control studies)	HIC (Japan, USA, Germany, Sweden, France, UK)	**.** Reduction in Road traffic crashes

In the study by Elder that assessed the effectiveness of ignition interlocks in reducing alcohol-impaired driving and alcohol-related crashes, no evidence of effectiveness was found in this intervention [51]. Although the findings were not statistically significant, Elder found that rates of single-vehicle night time crashes (SVNCs) were similar for first-time offenders with interlocks installed relative to those with suspended licenses (HR1.05, *p*-value = 0.85). This was lower for repeat offenders (HR0.46, *p* = 0.14). Investigators noted that the potential for interlock programs to reduce alcohol-impaired driving and alcohol-related crashes is currently limited by the small proportion of offenders who participate in the programs and the lack of a persistent beneficial effect once the interlock is removed [51].

Electronic stability control was also evaluated by Erke [56]. This is an active safety device for motor vehicles which aims to improve driving dynamics and to prevent accidents which result from loss of control. In this review, Erke found significant reductions in single vehicle accidents (− 49%; 95% CI: [− 55%; − 42%]), and smaller reductions in head-on collisions (− 13%; 95% CI: [− 17%; − 8%]). Similarly, multi-vehicle fatal accidents were also reduced (− 32%; 95% CI: [− 43%; − 20%]).

### Mixed interventions results

The mixed interventions categories consisted of systematic reviews which assessed the effectiveness of several interventions in reducing RTCs. In this category, there were three systematic reviews [36–38]. All three reviews were of critically low quality (See additional file 1). The reviews under this intervention are given in Table 9 below.

**Table 9:** Description of included studies: Mixed interventions

Review first author, date of publication	Date of search to identify studies for this review	Interventions	Participants	Number of included studies (types of studies)	Geographic area	Studied Outcomes
Bergen 2014 [37]	March 2012	Publicized sobriety checkpoint programs	All drivers	10 (Controlled before and after, Interrupted time-series)	HIC (USA, New Zealand)	Reduction in alcohol-involved crashes.
Lefio 2018 [38]	October 2016	Regulatory policies on alcohol consumption, educational and police enforcement	Pedestrians and drivers	41 (Time-series, RCT, population survey, systematic reviews)	HIC (USA, Ethiopia, Japan, Australia, Canada, Iran)	Reduction in Motor vehicle collisions as a result of regulating BAC.
Aguilera et al., 2014 [36]	December 2011	Engineering strategies Education strategies Law enforcement strategies	Pedestrians and drivers	22 (Before and After, Time series study with control group, Quasi-experimental study.	HIC (Australia, Canada, Spain, USA, France, Netherlands, England and Italy)	No reduction in Road traffic crashes

Bergen et al., [37] evaluated the effects of publicized sobriety checkpoint, media coverage, student designed social marketing campaign programs on alcohol involved crash fatalities. Results from this review indicated that eight out of 10 evaluations that measured alcohol involved crash fatalities reported reductions in the outcome after implementing publicized sobriety checkpoint programs. As such, publicized checkpoints were proven to be effective in preventing RTCs. Further, Bergen et al. found that stratified analysis of the effect of various factors on intervention effectiveness showed evidence of effectiveness for high-risk populations. However, differing check point configurations and publicized sobriety checkpoint programs were effective among high-risk populations of men aged 21–34 years and college students.

Lefio et al., [38] analysed several interventions which included monitoring motor carrier safety, regulatory compliance of trucking companies, a mandatory alcohol-testing program to reduce alcohol involvement in motor carrier crashes. Of all these interventions, the interventions which were found to reduce RTCs were blood alcohol content limit, enhancement of safety driving and driver standard. The results further indicated that among the working population, interventions most frequently shown to be effective were enforcement of national safety standards in the workplace (for companies that have transport operations) and interventions that used mandatory testing to prevent and severely restrict alcohol consumption.

Aguilera et al., [36] evaluated education as a behavior change strategy, as well as infrastructure interventions, inspections and other traffic safety policies. The studies included in the review focused on surveillance interventions. This intervention showed effectiveness in short-term assessments for example the points penalty system (SPP) was effective in promoting safe driving with outcomes more favourable to reducing morbidity and mortality. Enforcement was effective in changing driver’s behaviour, especially in relation to speeding and alcohol consumption associated with driving. Infrastructure development on the other hand promoted a safe environment, in which pedestrians, cyclists and drivers can live together. Finally, education was more informative and supportive of the other strategies used and did not present evidence of generating cultural change in road safety.

### ‘Other’ interventions

In the process of categorising these interventions, there were interventions such as vision screening for older drivers which did not fall in any of the interventions. Although these are targeted at individuals (drivers), we grouped these as other interventions. Three of the reviews [39, 40, 53] did not fall into any of the previous six categories. These interventions were then categorised as other interventions. Another review by Ditter et al.,[39] looked at the effect of designated drivers on alcohol related RTCs while Kwan and Mapstone [40] focused on how visibility aids used by cyclists and pedestrians can help increase visibility, reaction time and ultimately RTCs. The review by Kwan and Mapstone [40] included primary studies from high income countries as well as upper-middle income countries (South-Africa). A systematic review focusing on vision screening in older drivers by Desapriya et al., [53] did not find any studies that met the inclusion criteria and as such had 0 included studies and all these reviews are given in Table 10 below.

**Table 10:** Description of included studies: other interventions results

Review first author, date of publication	Date of search to identify studies for this review	Interventions	Participants	Number of included studies (types of studies)	Geographic area	Studied Outcomes
Ditter et al., 2005 [39]	July 2003	designated drivers	Drivers	8 (before and after, interrupted time-series)	HIC (USA and Australia)	**.** No reduction alcohol-related crashes was established.
Kwan and Mapstone 2006 [40]	Updated May 2009	All types of day-time and night-time visibility aids used on pedestrians/cyclists	Pedestrians and cyclists	35 (RCT)	HIC (USA, UK, Australia, Netherlands, Israel, Canada, Sweden, Finland Upper-middle (South Africa))	**.** Reduction in pedestrian and cyclist-motor vehicle collisions and injuries (fatal and non-fatal)
Desapriya et al., 2014 [53]	September 2006	Any screening method, including road and vision testing	Individuals Individuals over the age of 55 years with valid driver’s licenses.	0	N/A	**.** Reduction in traffic violations, crashes, motor vehicle injuries and fatalities

Ditter [39], evaluated the effects of specific designated driver programs which involved drivers moving long distances. However, no study that evaluated whether the use of designated drivers decreased alcohol related motor vehicle related injuries was found was found. Kwan and Mapstone [40] quantified the effect of visibility aids versus no visibility aids in pedestrians to reduce motor vehicle crashes and also to help drivers’ detection and recognition responses. Results of the review suggested that visibility aids influence drivers’ reaction, detection and recognition resulting in reduced RTCs. For daytime visibility, fluorescent materials in yellow, red and orange improved detection and recognition whereas in night-time visibility the use of lamps, flashing lights and retro reflective materials in red and yellow enhanced drivers’ detection and recognition. The review by Kwan and Mapstone [40] further indicated that the behaviors of drivers, pedestrians and cyclist in terms of intoxication and speeding are important issues to consider. Desapriya et al., [53] assessed the effects of vision screening interventions for older drivers who have problems with visual to prevent RTIs and fatalities, the review however did not find any study meeting the inclusion criteria.

## Discussion

Taking stock of existing systematic reviews is an important approach to use in informing both new research as well as policy and practice. This overview included 35 systematic reviews that evaluated different types of interventions to reduce RTCs. Out of all these reviews, 33 included studies which were conducted in HICs such as United Kingdom, Norway, Australia, Canada, Spain, USA, France, Netherlands, and Italy. Interventions assessed included enforcement, driver education, vehicle design, legislation, structural improvement and public awareness. Not all interventions showed consistency in reducing RTC’s. Sobriety check point and random breath testing, red light cameras, speed cameras, police patrols, roundabouts, streetlights and vehicle design improvement were consistently found to reduce RTCs.

This overview has provided evidence in HIC in which all the interventions have been applied. The overview has also established that the majority of the enforcement interventions (RLC, speed cameras, police interventions and sobriety check points) lead to a reduced number of road traffic crashes as compared to other intervention categories such as legislation and structural improvements. As indicated above, all the interventions which were assessed were implemented in HIC which include the UK, USA, England Australia, Germany, Denmark and Norway. It has been established from the descriptive analysis that enforcement programmes are very effective in reducing RTCs.

Other interventions that were falling in the public awareness only mass media campaigns were found to reduce the number of road traffic crashes and these results were in agreement with another study by Yadav [46]. These mass media campaigns must be encouraged as they educate school going children and the general public on safety measures when on the roads.

Interventions which were aligned to structural improvement strategies were not found to be very effective in preventing road traffic crashes. These interventions included street lighting, converting of intersections to round-about. In HIC, reducing blood alcohol content has proved to be effective in reducing road traffic crashes. The results from this overview is consistent with results found by Lefio [38] that blood alcohol concentration limit has a significance reduction in number of RTCs.

Our overview also confirmed the low number of reviews that have summarized evidence on interventions to prevent RTC on the African continent. This however, could have been as a result of few studies conducted which focused on interventions to prevent RTCs have been conducted on the African continent. This need to be explored so as to identify some of the effective interventions in road safety. In addition, there is need to synthesise evidence from LMICs through systematic reviews/meta-analysis where the majority of road traffic deaths occur.

Drivers are key individuals in these road traffic crashes and interventions that are driver-centered. The overview has also shown that programmes targeted on individuals/drivers are more effective than those targeted on road network and infrastructure. Some of these interventions include sobriety check points, class room sessions for drivers showed effectiveness in reducing RTCs. In summary, our findings are mapping to Fig. 1 in that we have identified interventions that are effective in road safety, these are mainly interventions which focused on behavioural change of drivers such as drink and driving, police presence and driver/pedestrian education.

### Overall completeness and applicability of evidence

The overview did not identify any systematic review focusing on LMICs. Caution should be taken when making inference on the effectiveness of some of these interventions especially in middle and low-income countries. The road infrastructure may greatly vary from developed countries where most of the primary studies included in the reviews took place.

Generally, the systematic reviews were of low quality. Twenty-two out of the thirty-five reviews were found to be either critically low or of low quality according to AMSTAR2 [12, 19, 22, 24–26, 30, 31, 33, 35–39, 43, 44, 46, 51, 56]. To assess the quality of the reviews, the reviewers read the methods section of the systematic reviews or searched for published protocols where possible. Most of the reviews lacked clarity on the methods used in the review process. Given the nature of some interventions, the majority of the reviews included studies that didn’t have comparison groups or of an observational design. This resulted in such reviews scoring poorly. This overview brought together findings from existing systematic reviews on interventions to prevent RTCs which can be used to inform future research and practice.

### Quality of the evidence

Most of the reviews included in this overview indicated that there was ‘weak level of evidence available’. Most of the systematic reviews conducted had included studies that were of poor quality in terms of study design, sampling etc. However, it was noted that, in general, some of the more recent studies were conducted with greater methodological rigour. A review by Goss et al., [28] observed that although increased police patrols appeared to reduce alcohol-related crashes and traffic fatalities in the identified studies, the quality and reporting of these studies was often poor. The review further found that there is need for methodologically rigorous research to evaluate effectiveness of these interventions.

Heterogeneity was present between included studies in the reviews. More evidence is needed to determine effectiveness of interventions such as, red light cameras, speed cameras, roundabouts, streetlights and vehicle design improvement in LMICs.

### Potential biases in the overview process

One of the potential biases in this overview could have resulted from the studies found during the search. In this regard, the search for potential systematic reviews did not comprehensively search for unpublished systematic reviews or grey literature. Unknown potential biases were minimised by following standard methods throughout the review. Two overview authors independently conducted eligibility assessment and data extraction, with resolution of conflicts through consultation with a third overview author.

## Conclusion

The review has revealed that individual based interventions have been found to be very effective as compared to other interventions. This finding suggests that the majority of the accidents are as a result of a driver’s behaviour. Therefore, results from the overview are anchoring on behaviour change to reduce RTCs. This change in behaviour can be done through sobriety check points, driver education, mass media campaigns for both drivers and other road users. In this vain, there is need for countries to strengthen interventions that target drivers, pedestrians, cyclists and motor cyclists. The overview has also established that there are very few reviews in Africa focusing on effectiveness of these interventions. Implications for new research is the need to conduct systematic reviews on effectiveness of interventions in LMIC.

## Supplementary Information

**Additional file 1: Table 1.** Methodological quality of systematic reviews.

**Additional file 2: Table 3.** Description of included systematic reviews in the overview: Enforcement category.

**Additional file 3: Table 11**. Search Strategy-Overview of systematic reviews.

## Data Availability

All data generated or analysed during this study are included in this published article (and its supplementary files). Extraction forms for all included systematic reviews are available from the corresponding author on reasonable request.

## Abbreviations

AIDS: Acquired Immunodeficiency Syndrome
BAC: Blood Alcohol Concentration
BIGRS: Bloomberg Initiative for Global Road Safety
CI: Confidence Interval
DCT: Digital Countdown Timer
DD: Drinking and Driving
DUI: Driving under the Influence
ESC: Electronic Stability Control
GDL: Graduated Driver Licensing
HIC: High Income Countries
HIV: Human immunodeficiency virus
HR: Hazards Ratio
IQR: Inter-quartile range
LMICs: Low- and Middle-Income Countries
NRSI: Non-randomized Studies of therapeutic Interventions
PICO: Population, Intervention, Comparison and Outcome
RBT: Random Breath Testing
RCT: Randomized Controlled Trials
RDD: Ridding with Drinking Drivers
RLCs: Red Light Cameras
RoB: Risk of Bias
RTC: Road Traffic Crash
RTCs: Road Traffic Crashes
RTIs: Road Traffic Injuries
SBT: Selective Breath Testing
SPP: Points Penalty System
SVNCs: Single Vehicle Night Time Crashes
UK: United Kingdom
USA: United States of America
WHO: World Health Organization
